# Climate change and health – what’s the problem?

**DOI:** 10.1186/1744-8603-9-4

**Published:** 2013-02-09

**Authors:** Matthew HR Anstey

**Affiliations:** 1 Lecturer in Anesthesia, Harvard Medical School, Boston, MA, USA; 2 Harkness Fellow in Health Policy, Commonwealth Fund, New York, USA

## Abstract

The scientific consensus is that global warming is occurring and is largely the result of greenhouse gas emissions from human activity. This paper examines the health implications of global warming, the current socio-political attitudes towards action on climate change and highlight the health co-benefits of reducing greenhouse gas emissions. In addition, policy development for climate change and health should embrace health systems strengthening, commencing by incorporating climate change targets into Millennium Development Goal 7.

## Introduction

The final report of the Lancet commission on climate change suggested that “climate change is the biggest global health threat of the 21^st^ century” [1]. In a medical world that is oft dominated by ‘evidence based medicine’, there is resounding scientific consensus that global warming is occurring and is largely the result of greenhouse gas emissions from human activity [2]. And yet, while political accord on tackling global warming remains fractious, global carbon dioxide emissions continue to rise, hitting a record high last year [3]. Within this landscape, this commentary outlines the health implications of global warming, the current socio-political attitudes towards action on climate change, and argues that policy development for public health and climate change should highlight the health co-benefits of reducing greenhouse gas emissions.

### Health implications

In the last 3 decades the Earth’s surface has warmed by approximately 0.6°C and climate models predict further rises between 1.1°C and 6.4°C over the 21^st^ century [4]. These rising temperatures will be responsible for increased number of mosquitoes (models suggest that 300 million more people will be affected by malaria by 2080), heatwaves causing heatstroke, and increased dengue fever cases, gastroenteritis and other climate sensitive infectious diseases [5]. World Health Organisation (WHO) estimates in 2000 suggested that 5.5 million DALYs were lost as a consequence of increased cardiovascular disease, diarrhoea, malaria, injuries from flooding and malnutrition due to climatic changes [6]. Their assessment excluded the ‘unquantifiable’ health consequences from pollution, changes in food production, temperature extremes, population displacement and conflict. However, other groups have suggested that crop yields will fall by 20-40% with rising temperatures, exacerbating existing food shortages that already lead to malnutrition and the deaths of 3.5 million women and young children every year [7]. Similarly, 16% of people in developing countries currently do not have access to clean water, and 48% lack access to adequate sanitation, a situation that will deteriorate further with changing rainfall patterns [8]. Major weather related disasters that have led to more than 2 billion people being affected by floods, drought, fires and cyclones over recent years are also expected to increase [9]. Natural disasters lead to mass environmental disruption, displacement and migration of people, and advancing the process of urbanisation. Urbanisation itself, with its higher density population, potentially increases people’s vulnerability to climate change, especially when settlements are not designed to be ‘climate resilient’ [1]. All of these considerations exclude the potential indirect health consequences from the economic disruption that climate change will bring, especially for developing countries with agrarian economies.

A criticism regarding predictions of global warming and health is the uncertainty and complexity of attributing increased disease burden to global warming. However, the uncertainty is not that global warming will be responsible for exacerbating the global public health problems of poverty, infectious and non-communicable diseases, but by how much. On a scientific level, estimating the health effects of climate change is difficult given the absence of a clear comparison group, the long lag time between changes and effects and the myriad of potential outcomes from climate change and the large number of non-climatic influences on these outcomes. This uncertainty has been successfully exploited in dampening the call to action.

### Equity implications

Article 3 of the United Nations Framework Convention on Climate Change (UNFCCC) states “The Parties should protect the climate system for the benefit of present and future generations of humankind, on the basis of equity and in accordance with their common but differentiated responsibilities and respective capability” [10]. This touches on the assertions of developing countries that the historical responsibility for climate change rests with the developed world and that they have other more pressing needs for their limited resources. The health risks of climate change are concentrated in the poorest countries, which have the least adaptive capacity to deal with them, while the majority of greenhouse gases originate mainly from developed countries (the poorest 1 billion in the world contribute only 3% of the global footprint).

The current world population of close to 7 billion is expected to reach 10 billion by 2100, putting extra stress on already struggling health systems and cities, and exacerbating food and water insecurity [11]. Much of the increase in the population is expected to come from the ‘high fertility’ countries (predominantly developing countries in Asia and Africa). The ‘underdeveloped countries’ rely on high fertility rates to combat high infant mortality, lack of social security and a reliance on agriculture. Until these issues are addressed, they feel it is unfair to blame them for their population growth. However in looking to the future, modeling suggests that by 2035, energy-related carbon dioxide emissions in non-OECD countries (28.2 billion metric tons) will be almost twice the level of those in OECD countries [12]. In creating an equitable response to climate change, this then raises two possibly conflicting actions. Poor countries need economic development to provide them with the means to adapt to climate change, but at the same time, global carbon emissions must be reduced. This has given rise to the concept of ‘contraction and convergence’ whereby developed countries need to reduce their emissions (and forgo luxury goods such as air travel) while allowing developing countries to increase theirs [13].

### Barriers to change

#### Framing

Framing “[w]hether the planet is warming” as ‘climate change’ or ‘global warming’ appears to affect how people understand, or believe, whether this phenomenon is occurring [14,15]. UK research has shown that there is widespread awareness of climate change as a concept, but it takes low priority as an issue, and only a minority of people attempt to reduce their energy usage [16]. The reasons put forward by individuals are similar to those heard in other countries, and strikingly similar to those heard on a governmental level, and include: blaming others (“if the USA isn’t doing anything, why should I?”); faith in technological solutions; or pointing to other issues of supposed greater importance [16,17]. These cognitive defences create a significant obstacle to societal action on climate change.

#### Theoretical approaches

The policy debate around efforts to tackle global warming is divided into either mitigation or adaptation responses. Mitigation refers to the efforts to slow or even reverse climate change by reducing greenhouse gas emissions. Some people would argue that we should focus on preventing the cause of global warming, whereas others point to the fact that global warming is already occurring and that we must learn to face its consequences. While primary prevention is a central tenant of public health, policies for climate change mitigation must come largely from other sectors, such as energy and transportation. Work on finding a way for these sectors to collaborate (which usually work in isolation) is a challenge. More conventionally, public health has focused on preparing to face the impacts of climate change (adaptation). As a discipline, public health more easily relates to adaptation, and much of the efforts have revolved around preparedness developments. However the focus on mitigation takes on a global focus, whereas the consequences of climate change have regional implications and thus adaptation solutions tend to be state-based.

Currently, economic and institutional approaches make up the majority of efforts to curb emissions [18]. By placing a price on the negative externality of carbon emissions, economic believers think that this will resolve the market failure that leads to climate change. Others aim to work on improving intergovernmental cooperation and strengthening institutions to lead to collective efforts. However, these approaches cannot exist in isolation, and while acting on climate change makes economic sense in the long term, as any credit card company will tell you, people would prefer to pay later.

#### Public opinion, individual countries and NGOs

The support for action on climate change, more in principle than action, is reflected in the public opinion in the US. Recent surveys showed confusion amongst the public in just how significant they believe global warming to be. 58% of people surveyed believed that recent natural disasters were evidence of global warming and 62% of people felt that climate change was ‘already a big problem and we should be leading the world in solutions’ [19,20]. Despite this, there is less support for how to finance action on climate change, with a majority of people feeling that a carbon tax should not be adopted, as there are more important problems to tackle [21]. When prioritising aid distribution to developing countries, people placed climate change fourth, behind measures such as improving education, reducing poverty and responding to natural disasters [22].

In addition to the intergovernmental approach, there are many countries and NGOs that have started their own climate change responses. In 2005 the European Union began the implementation of its Emissions Trading Scheme [23]. In the most recent Chinese five-year plan, they introduced the goal of ‘gradually establishing a carbon trade market’ by a 16% reduction in energy and carbon intensity and increasing non-fossil energy to 11.4% of total use [24]. US business (and the government) has traditionally responded to a call for a mandatory climate policy by saying that it would reduce US competitiveness. Any climate policy would present costs for US firms regardless what happens in other countries, but both China and Europe have invested more than double the US in clean energy investments (US $18.6b 2009), recognising the potential for energy independence and an expanding market [25].

The major NGOs approach to climate change and health reflects their broader approaches to global health delivery. For example the Gates Foundation states that while they believe climate change to be a major issue and they do fund some projects designed to help adaptation, they do not fund mitigation efforts [26]. Organizations such as the Gates Foundation or the Global Fund see that the wider task of climate change mitigation is the role of government and not compatible with their business model approach to health improvement. On the contrary, organizations such as Oxfam and the International Red Cross have specifically designated global warming adaptation and prevention strategies as an integral part of their missions.

#### International political situation - UNFCC

Much of the policy development in climate change has come from attempts at international consensus building by the 194 parties to the UNFCCC who share a long-term vision of limiting global warming [27]. In December 1997, the Kyoto protocol was adopted and since then has been ratified by 192 bodies. Under the provisions of the protocol, 37 countries and the EU have committed to reducing greenhouse gas emissions by an average of 5% over the 1990 levels by 2012, but the largest emitter of greenhouse gases (the USA) is not a signatory to the protocol [28].

Unfortunately in the years since the Kyoto international negotiations have been fractured, and outcomes have been non-binding. Prior to the 2011 Durban conference, the US Special Envoy on Climate Change Todd Stern acknowledged that for the US to act, they would want all the major emitters (China, India, Brazil and Russia) taking on the same binding obligations, and it doesn’t appear that countries were willing to enter into this [29]. The Durban conference re-established commitment to a second commitment period under the Kyoto protocol, and creating the ‘Durban platform’, a legally binding treaty to address global warming that includes the developing superpowers of China and India, as well as the US [30]. However the terms of the treaty are to be determined by 2015, with commencement in 2020, a result that permits ongoing negotiation and discussion, without binding force. Many governments expressed a loss of trust in the negotiation process, a feature that has characterized UN climate talks [31]. Media reports of the recent Bonn climate talks highlighted this mistrust, with suggestions of a re-opening of the ‘rich-poor’ divide [32]. This division is that developing countries have accused the US and the EU of trying to avoid making deeper emissions cuts and funding to help poorer nations cope with climate change. Any international agreement requiring more equitable emission levels would require the West to constrain their energy-intense lifestyle. With the depressed global economy, it appears that countries are playing a prisoner’s dilemma game, with a potential better outcome for all, but no-one willing to act alone.

### What can be done?

#### Call it pollution

Pollution itself has significant health consequences, being linked to cardiorespiratory health [33,34]. Given the slow rate of progress in climate change adaptation for health reasons, it may be better to ‘frame’ the consequences of emissions with regards to pollution. Success can be found in the US Clean Air Act (and amendments) which has made significant improvements in air pollution over the decades [35]. As a framing issue, addressing carbon emissions as pollution may be a more easily understandable message [36]. Focusing on ‘win-win’ solutions in both developed and developing countries would combat the arguments of climate sceptics. For example, changing the infrastructure of cities to reduce reliance on cars and increase exercise will help the fight against obesity. Decommissioning coal-fired power stations will reduce the air pollution that is responsible for millions of deaths from cardio-respiratory diseases [37].

#### Quantitative research

From a public health research perspective it is important to have the scientific research to facilitate policy change and advocacy work. WHO is currently revising its global burden of disease assessment, which will update the statistics on the health implications of climate change. This will provide further weight for the need for action (similar to the Stern report). However, as recently pointed out, there remains a relative lack of quantitative research in climate change and health, with significant gaps in adaptation research and publications from developing regions [38]. Regional health impact assessments to identify climate-sensitive health outcomes, predict future scenarios and estimate the attributable burden to climate change would be helpful in crafting policy solutions [39].

#### Adaptive management

Similar to quality improvement methods, ‘adaptive management’ has been suggested as one approach to practically respond to climate change [40]. This approach identifies potential collaborators, determines each stakeholders’ interpretation of causes and options for management, models the potential consequences of different choices, and evaluates and modifies the public health intervention based on the lessons learned. The ability to adapt to a shifting landscape is the key to this approach, as it incorporates a way of responding to both the uncertainty of the extent and magnitude of climate change, and societal and political opinions.

Many of the components of adaptation are not dissimilar to those that are needed, and already being done, to improve health. Poverty reduction, improving water and sanitation, improving health infrastructure are essential for giving people in developing countries a chance at enduring the potential threats they will face from climate change. A policy solution that I would propose is linking climate change to the Millennium Development Goals. The current and anticipated effects of global warming threaten progress on each of the MDGs and despite their limitations they are widely accepted targets. The seventh goal “to ensure environmental sustainability” has no mention of climate change. Integrating the target of reducing greenhouse gas emissions into MDG 7 would be help to achieve the MDGs and to build upon the inter-governmental and inter-organizational efforts already taking place [41].

Finally, it is clear that while technological challenges exist (no effective vaccines for many of the climate sensitive infectious diseases (such as malaria, dengue fever), the need for agricultural innovation and increased food productivity and improved green and disaster resilient housing) many of the problems that climate change raises could be tackled through resources that we already have available. Development work needs to address the current fragmentation of health systems, especially in developing countries, to provide health systems capable of delivering reliable clinical services and responding to climate induced threats to health. Any strategy needs to have a combination of local, national, regional and global components, with input from academics, health professionals, NGOs, communities and governments to act on a truly global problem. Rather than think of this as a challenge, perhaps climate change is the impetus that is needed to unite policy makers in considering the social determinants of health.

## Conclusion

The corroboration of scientific evidence across disciplines has confirmed that global warming is occurring and the potential consequences are frightening. It is time for public health advocates to draw on their past successes in tackling the health consequences of pollution, and to draw the link between the causes of global warming and pollution. In addition, strategies that link stakeholders and current development goals and provide feedback data from climate change adaptation and mitigation approaches are needed as we move forward to face “the biggest global health threat of the 21^st^ century” [1].

## Competing interests

The author declares that he has no competing interests.

## Authors’ contributions

MA conceived of the article, drafted and approved the final manuscript.
